# Identification of Novel QTL for Mercury Accumulation in Maize Using an Enlarged SNP Panel

**DOI:** 10.3390/genes15020257

**Published:** 2024-02-19

**Authors:** Jionghao Gao, Jianxin Li, Jihong Zhang, Yan Sun, Xiaolong Ju, Wenlong Li, Haiyang Duan, Zhengjie Xue, Li Sun, Javed Hussain Sahito, Zhiyuan Fu, Xuehai Zhang, Jihua Tang

**Affiliations:** 1 Key Laboratory of Wheat and Maize Crops Science, College of Agronomy, Henan Agricultural University, Zhengzhou 450002, China; gao13598069385@126.com (J.G.); bnmvbncvb@163.com (J.L.); 18348062126@163.com (J.Z.); sunyan15938845165@126.com (Y.S.); juxiaolog@163.com (X.J.); lwl15737132455@163.com (W.L.); 18839046763@163.com (H.D.); xuezhengjie2023@163.com (Z.X.); sun_lier@126.com (L.S.); javedhussain@henau.edu.cn (J.H.S.); fuzhiyuan2004@163.com (Z.F.); 2 The Shennong Laboratory, Zhengzhou 450002, China

**Keywords:** mercury accumulation, maize tissue, genetic loci, genome-wide association analysis

## Abstract

Mercury (Hg) pollution not only poses a threat to the environment but also adversely affects the growth and development of plants, with potential repercussions for animals and humans through bioaccumulation in the food chain. Maize, a crucial source of food, industrial materials, and livestock feed, requires special attention in understanding the genetic factors influencing mercury accumulation. Developing maize varieties with low mercury accumulation is vital for both maize production and human health. In this study, a comprehensive genome-wide association study (GWAS) was conducted using an enlarged SNP panel comprising 1.25 million single nucleotide polymorphisms (SNPs) in 230 maize inbred lines across three environments. The analysis identified 111 significant SNPs within 78 quantitative trait loci (QTL), involving 169 candidate genes under the Q model. Compared to the previous study, the increased marker density and optimized statistical model led to the discovery of 74 additional QTL, demonstrating improved statistical power. Gene ontology (GO) enrichment analysis revealed that most genes participate in arsenate reduction and stress responses. Notably, *GRMZM2G440968*, which has been reported in previous studies, is associated with the significant SNP chr6.S_155668107 in axis tissue. It encodes a cysteine proteinase inhibitor, implying its potential role in mitigating mercury toxicity by inhibiting cysteine. Haplotype analyses provided further insights, indicating that lines carrying hap3 exhibited the lowest mercury content compared to other haplotypes. In summary, our study significantly enhances the statistical power of GWAS, identifying additional genes related to mercury accumulation and metabolism. These findings offer valuable insights into unraveling the genetic basis of mercury content in maize and contribute to the development of maize varieties with low mercury accumulation.

## 1. Introduction

Mercury (Hg) pollution poses a significant threat to both the environment and plant growth. Hg is highly toxic and can accumulate in the food chain through methylation, bioaccumulation and biomagnification, potentially affecting animal and human health [1,2]. As a major source of food, industrial materials and livestock feed, maize plays a crucial role in global food production and human well-being. However, increasing industrial activities, automobile usage and pesticide applications have led to rising emissions of Hg gas and particles into the atmosphere. This atmospheric Hg eventually contaminates soil through deposition [3,4,5]. Maize cultivation on contaminated soils introduces risks, as Hg can accumulate in plant tissues and enter the food chain [6]. Inside plants, Hg disrupts critical physiological processes like water and nutrient uptake, transpiration, and photosynthesis. It also impairs enzyme activity, slowing growth and decreasing biomass production, and can even cause mortality [7]. The adverse effects of Hg toxicity extend to humans. Accumulation of Hg in vital organs such as the kidneys and central nervous system has been linked to harmful impacts [1,8]. Understanding the genetic basis of Hg accumulation in maize and developing varieties with reduced Hg uptake are essential strategies for mitigating these threats to both plant and human health.

Recent years have seen a growth in genetic studies investigating Hg accumulation mechanisms. For instance, quantitative trait locus mapping has identified regions associated with Hg tolerance in rice and maize [9,10]. Overexpression analyses have revealed genes that enhance Hg accumulation and tolerance in plants like rice, Arabidopsis and poplar [11,12,13]. These findings provide valuable insights but also highlight the complexities of Hg accumulation in plants. Further genetic investigations are needed to better understand this process. GWAS, utilizing linkage disequilibrium (LD), have emerged as a powerful tool for dissecting genetic–phenotypic relationships. Compared to traditional linkage mapping, GWAS allows the analysis of broader, more diverse populations with higher marker densities, improving mapping resolution to the single-gene level [14]. Maize is well suited for GWAS due to its rapid LD decay and abundant diversity [15,16]. The first maize GWAS in 2007 examined 8590 loci in 553 elite maize inbred lines to identify genes related to oleic acid content in maize kernels [17]. Subsequent studies have identified loci impacting oil concentration or fatty acid composition in kernels and identified QTL related to arsenic content in various maize tissues [16,18].

The statistical power of GWAS depends on various factors, including population size, diversity, marker density, and the choice of statistical models. By optimizing these aspects, more genetic determinants of target traits can be identified [19]. Studies have demonstrated that analyzing large germplasm collections with high-density molecular markers can successfully identify genetic loci [20]. Appropriate statistical models facilitate a more comprehensive resolution of the genetic structure and relationships [21]. The GWAS method SUPER augmented power through model optimization [22]. Enhancements in sequencing depth and marker density facilitate improved genomic coverage, enabling the identification of both genes and non-genetic contributions to traits [23]. Dissecting additional maize traits using an enlarged panel revealed further loci [24]. Advancements in sequencing technology, reduced costs, and continuous GWAS algorithm improvements have made it an efficient approach for identifying genome–phenotype associations and genetic bases of complex traits [25,26].

In this study, we re-conducted a GWAS to dissect the genetic basis of Hg content in five tissues of the 230 maize inbred lines [27]. Our aim was to leverage an expanded SNP panel and optimized statistical models to identify novel loci governing Hg levels in different tissues, enhancing understanding of this complex trait. Overall, characterizing the genetic basis of Hg accumulation in maize has important implications for breeding varieties with reduced Hg uptake and enhancing food safety and human health.

## 2. Materials and Methods

### 2.1. Plant Materials and Field Trials

An association mapping panel (AMP) of 230 diverse maize inbred lines, encompassing temperate, tropical, and subtropical backgrounds, was utilized. The panel consisted of 151 inbred lines from temperate backgrounds and 79 from tropical/subtropical backgrounds. In 2012, the AMP underwent cultivation at two distinct locations: Xixian (XX, 114°72′ E, 32°35′ N) and Changge (CG, 113°34′ E, 34°09′ N) are located in northern China. XX experienced an average temperature of 15.2 °C and rainfall of 873.8 mm, while CG had an average temperature of 14.3 °C and rainfall of 462.8 mm, following a randomized complete block design with three replications. These locations were chosen specifically for sampling and evaluating arsenic (As) and mercury (Hg) content. The AMP had previously been employed in a GWAS study exploring the genetic basis of As and Hg accumulation in five maize tissues [27]. Notably, the soil at XX and CG exhibited Hg contents of 457.57 ± 31.30 μg kg^−1^ and 345.40 ± 22.24 μg kg^−1^ (pH 6.5), respectively. Detailed information on maize inbred lines, trial specifics, and field management practices can be found in a previous study (Supplementary Table S1, [27]).

### 2.2. Measurement of Hg Content

Hg content was collected from the kernel, axis, stem, bract, and leaf of the AMP at both XX and CG locations. The phenotypic measurement, as described previously [27], utilized the same dataset for the current GWAS. To facilitate the GWAS, each combination of tissue (kernel, axis, stem, bract, and leaf) at each location (XX, CG, and BLUP) was treated as an individual phenotypic variable. For instance, “Axis_CG” represents the Hg content in the axis at the Changge location. In total, we obtained 15 phenotypic variables, including 10 for the five tissues at XX and CG locations and 5 representing the BLUP value of each inbred line across the two locations.

### 2.3. Genotype and GWAS

In this study, the AMP comprises 513 maize inbred lines extensively used in prior studies [15]. To augment genotyping data, 556,809 SNPs (referred to as 0.55 M) with a minor allele frequency (MAF) above 0.05 were derived from this set of 513 lines. This dataset combines 56,110 SNPs from 513 lines and 1.03 million SNPs from 368 lines, a subset of the 513 lines. The integration of these datasets used a two-step imputation method based on identity by descent (IBD) and k-nearest neighbors (KNN) algorithms, as described previously [24]. Notably, utilizing 0.55 M has demonstrated a significant enhancement in statistical power in GWAS for traits such as oil concentration and tocopherol content when compared to low-density markers like 56,110 SNPs [28]. Additionally, a second genotype dataset, comprising 1.25 million SNPs (referred to as 1.25 M) with a MAF ≥ 0.05, was obtained through an effective imputation method integrating data from four genotyping platforms [29], including Illumina Maize SNP50 BeadChip [30], deep RNA-sequencing [31], genotyping by sequencing (GBS) and Affymetrix Axiom Maize 600 K array [32]. This high-density genotype dataset has been successfully used in previous GWAS studies elucidating the genetic basis of traits such as oil concentration, amino acids, and arsenic content in maize kernel and other tissues [18,33,34]. Both genotype datasets, 0.55 M and 1.25 M, were employed in the subsequent GWAS analysis of the current study, and they are available at http://www.maizego.org/Resources.html (accessed on 18 February 2024).

GWAS of the 15 variables in the 230 inbred lines underwent analysis in several steps:

(1) Multiple statistical models, namely 5PCs + K, Q, K, and Q + K, were employed. The 5PCs + K model controls both the top five principal components (PCs) and the kinship matrix, as used in the study by Zhao et al. [27]. The Q model controls only the population structure, the K model solely controls the kinship matrix, and the Q + K model controls both the population structure and the kinship matrix. These analyses utilized the 0.55 M genotypic datasets, and the optimal statistical model was chosen based on quantile–quantile (QQ) plots.
(2) GWAS results under 0.55 M were compared between 5PCs + K and the optimal statistical model to assess whether changing the model could enhance the statistical power of GWAS.
(3) Under the optimal statistical model, GWAS was conducted using an enlarged genotypic dataset (1.25 M). The results from GWAS with the 0.55 M and 1.25 M datasets were compared to determine if increasing the marker density improved the statistical power of the analysis.
(4) Finally, the GWAS results of the optimal statistical model under the enlarged 1.25 M genotypic dataset were used for subsequent analysis. The effective marker number (En) of the two genotypic datasets was calculated using GEC V1.0 software. The result indicated that En was 490,548 for the 1.25 M dataset and 250,345 for the 0.55 M dataset. In addition, the software suggested a significance threshold of *p* ≤ (1/En), which was used for the association analysis.

### 2.4. Candidate Gene Identification

In preceding studies, we employed a dataset of 1.25 million SNPs to investigate the linkage disequilibrium decay among 500 maize inbred lines. The results revealed an LD decay of approximately 30 kilobases (kb) with an R^2^ value of 0.1. Consequently, a 30 kb interval around both the upstream and downstream regions of a significant SNP marker’s physical position was defined as a locus. For gene identification within these identified loci from GWAS, we utilized the B73 reference genome (RefGen_v2) and obtained the maize whole-genome gene list from the Maize Genetics and Genomics Database (MaizeGDB, http://www.maizegdb.org, accessed on 18 February 2024). This list was employed to search for candidate genes within each significant locus. The selection of the most likely candidate gene at each locus was based on functional annotations and expression profiles of the genes in different tissues of the maize inbred line B73.

### 2.5. Analysis of Expression Level Association of Candidate Genes

In our study, we performed an analysis to explore the association between gene expression levels and genetic variation (1.25 M) using expression data from kernels collected 15 days after pollination in the 368 maize inbred lines. This analysis aimed to identify expression quantitative trait loci (eQTL), which are specific genetic loci associated with variations in gene expression. To assess the significance of these associations, we applied a stringent criterion, considering associations as significant only if they met the condition of *p* < 1/En, where En represents the effective marker number. This criterion ensures that only robust and biologically relevant associations between genetic variants (SNPs) and gene expression levels are deemed significant eQTL.

### 2.6. Haplotype Analyses of Candidate Genes

Based on the study’s results, we performed a thorough screening of all single nucleotide polymorphisms (SNPs) within candidate genes using genotype data. Haplotypes were formed by combining best linear unbiased prediction (BLUP) values from the 230 inbred lines. Specifically, haplotypes with representation by more than eight inbred lines were systematically chosen, and their differences were rigorously examined through a *t*-test, with a significance threshold set at *p* < 0.01. Additionally, we meticulously generated a heatmap illustrating pairwise linkage disequilibrium using the “LDheatmap” package in the R 3.6.0 statistical software [35].

## 3. Results

### 3.1. Model Comparison and Selection

**Figure 1:**
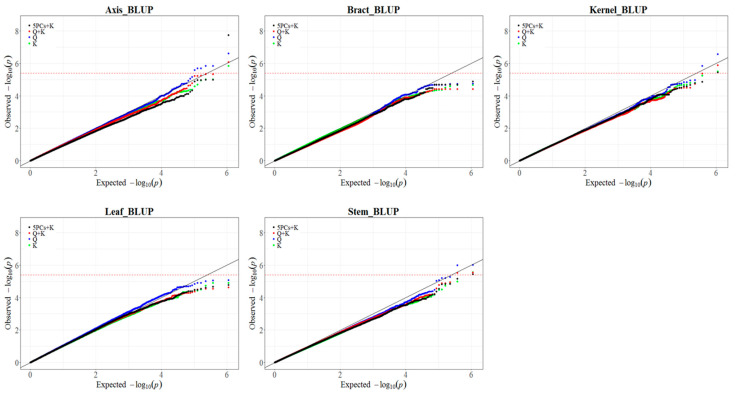
Comparison of Quantile–Quantile (QQ) plots resulting from GWAS. QQ plots were generated based on 0.55 M SNPs using four models (Q, K, Q + K and 5PCs + K) for BLUP values of mercury content in maize axis, stem, bract, leaf, and kernel.

Previous research investigated the genetic factors influencing Hg content in various maize tissues. However, limitations were identified in the statistical model used (5PCs + K), which exhibited excessive conservatism, leading to reduced detection of true associations (type II errors). Additionally, the significance threshold for declaring SNP-trait associations was relatively lenient [36]. To address these limitations, we reanalyzed the Hg content using different GWAS models and an enlarged SNP panel. We first analyzed the Hg content using four statistical models (Q, K, Q + K, 5PCs + K) with 0.55 million SNPs. Examination of quantile–quantile (QQ) plots revealed that the Q model consistently had the best fit to the data (Figure 1 and Figure S2). Comparing results from the Q and 5PCs + K models affirmed the improved power of the Q model (Supplementary Figure S1). Using a Bonferroni-corrected significance threshold (*p* ≤ 1/EN_1_, EN_1_ = 250,345), the Q model identified 46 significant SNPs associated with 32 QTL, whereas 5PCs + K identified only three QTL (all also detected by Q). Similarly, the Q model is also found to be superior to other models at 1.25 M (Supplementary Figure S3).

### 3.2. Boosting GWAS Power through Increased Marker Density

**Figure 2:**
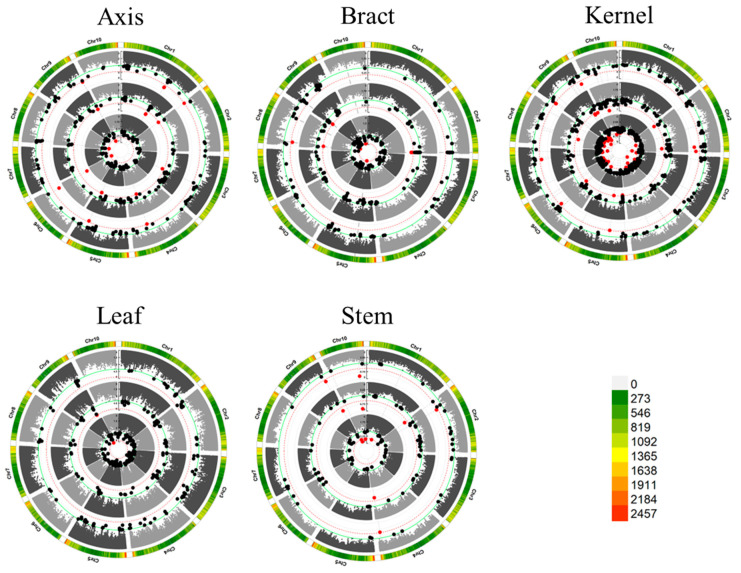
Manhattan plots for mercury content based on 1.25 M SNPs in five different tissues across different locations. Significant loci are highlighted in red and black. The red locus indicates significance at *p* ≤ 2.0 × 10^−6^ (EN_2_ = 490,548), while the black locus corresponds to significance reported in a previous study at *p* ≤ 1.0 × 10^−4^. The threshold lines are colored in red and green, respectively. The ring represents CG, XX and BLUP from the inside out.

We then investigated whether increasing the marker density could further improve power. Re-running GWAS with 1.25 million SNPs under the Q model revealed more significant SNPs than with 0.55 million SNPs (Supplementary Figures S4 and S5). Specifically, 1.25 million SNPs identified 111 significant SNPs associated with 78 QTL at (*p* ≤ 1/EN_2_, EN_2_ = 490,548, Figure 2), versus 26 SNPs and 22 QTL with 0.55 million SNPs. These findings demonstrate that increasing the marker density augments GWAS power to discover novel trait-associated loci.

### 3.3. Significant Loci and Tissue-Specific Variability

**Figure 3:**
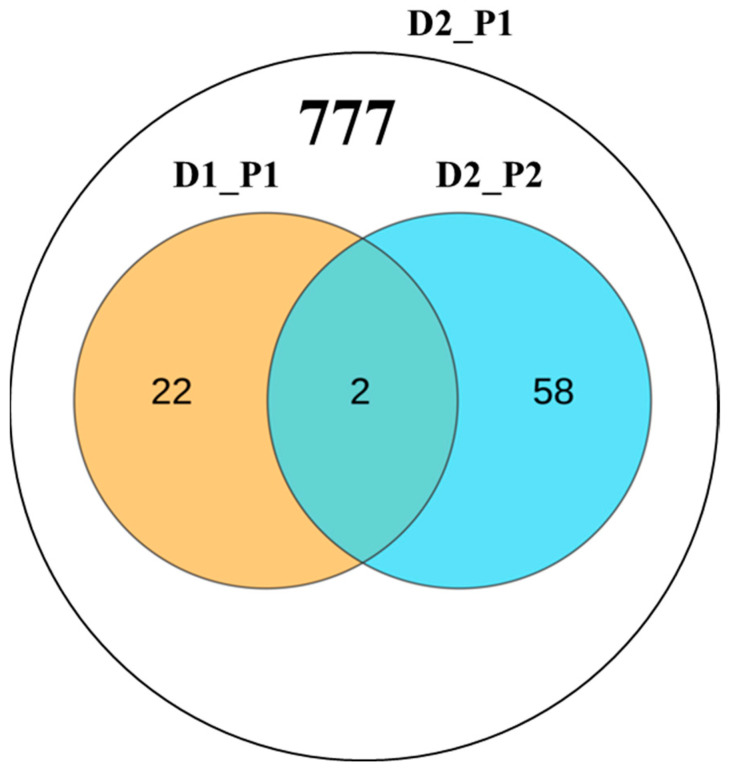
GWAS discoveries for two genotypic datasets with different numbers of markers. A Venn diagram depicts significant loci identified in the two genotypic datasets along with their respective thresholds. Dataset 1 (D1) comprises 0.55 million SNPs, while Dataset 2 (D2) contains 1.25 million SNPs. P1 and P2 represent the significance thresholds for *p* ≤ 1/NE, where NE is 250, 345 for D1 and 490, 548 for D2.

To assess the impact of increased marker density on GWAS detection efficiency, we conducted an analysis at a family-wise error rate of 0.05. In Dataset 1, we identified 24 non-redundant QTL (*p* ≤ 4.0 × 10^−6^, EN_1_ = 250,345) and in Dataset 2, we identified 61 non-redundant QTL (*p* ≤ 2.0 × 10^−6^, EN_2_ = 490,548). Only two loci were common between the two datasets. When the *p*-value was set to *p* ≤ 4.0 × 10^−6^, consistent with Dataset 1, a total of 777 QTL were identified, including 24 non-redundant QTL from Dataset 1 (Figure 3).

Expanding our analysis to different tissues revealed 111 SNP-trait associations across 78 loci categorized into 61 non-redundant QTL at a significance level of *p* ≤ 2.0 × 10^−6^ (Table S2). Within the CG environment, 50 SNPs involving 34 loci were identified, each explaining phenotypic variation ranging from 8.38% to 24.30%. Notably, three significant SNPs associated with axis tissue were discovered across three QTL, explaining phenotypic variance ranging from 10.3% to 24.3%, with a mean of 16.8%. Similar findings were observed for bract, kernel, leaf, and stem tissues. In the Xixian environment, 37 SNPs involving 25 loci were identified, each explaining 8.47% to 24.71% of the phenotypic variation. For each tissue, specific SNPs and QTL were discovered, contributing to our understanding of the genetic basis of these traits. In the BLUP environment, 24 SNPs and 19 loci were identified, each explaining 9.70% to 23.27% of the variance. Comprehensive analysis across distinct tissues and locations enriched our understanding of the diverse landscape of significant loci, highlighting the genetic contributions to these traits.

### 3.4. Colocalized Loci Identified across Various Tissues and Environments

**Figure 4:**
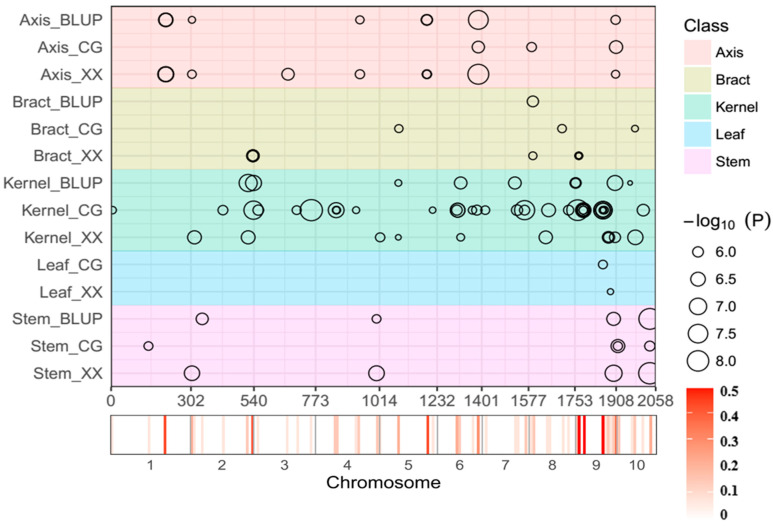
Chromosomal distribution of QTL for Hg content identified by GWAS. QTL positions and significance (represented by circle size) across the maize genome are indicated by black circles. The x axis displays physical positions across the maize genome in Mb. A heat map below the x axis illustrates the density of QTL across the genome, with a window size of 10 Mb. Detailed information on all detected QTL is provided in Table S2. Different traits are marked by distinct colors as shown on the right.

Notably, we identified 15 non-redundant QTL encompassing 36 SNP-trait associations consistently detected across tissues and environments (Figure 4). Particularly noteworthy were non-redundant QTL associated with stem tissue housing three QTL within a 127.32~127.37 Mb interval on chromosome 10. These QTL were consistently identified in all environments, explaining sizable phenotypic variations. Another non-redundant QTL linked to axis tissue appeared in three environments within a 155.63~155.69 Mb region on chromosome 6. The observed co-localization patterns indicate stability of these loci against environmental influences.

### 3.5. Functional Analysis of Identified Genes

**Figure 5:**
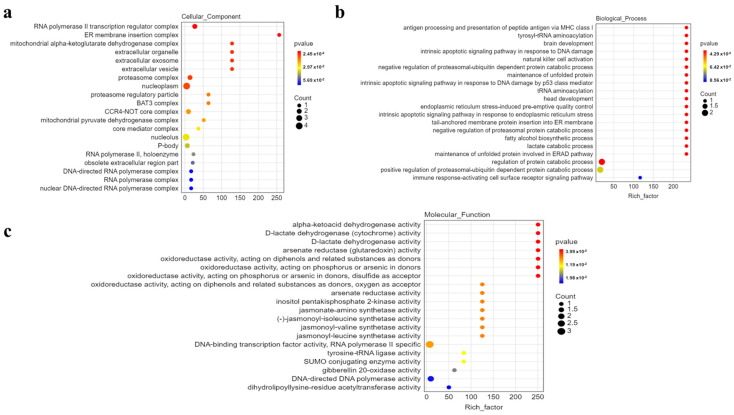
Enriched GO terms among the candidate genes. Enriched GO terms in (**a**) biological process; (**b**) molecular process; (**c**) cellular process.

To pinpoint the most likely candidate gene and understand their molecular functions. Subsequently, we conducted gene ontology (GO) analysis to reveal the functions served by these genes (Figure 5). Our analysis revealed that these genes predominantly cluster around essential molecular activities, including DNA binding, arsenate reductase (glutaredoxin) activity, jasmonate-amino synthetase activity and other functions. This GO analysis provides valuable insights into the molecular functions of these genes, shedding light on their involvement in the mechanisms of resistance to metal stress and significantly enhancing our understanding of their contributions to plant biology.

### 3.6. Candidate Gene Analysis

**Figure 6:**
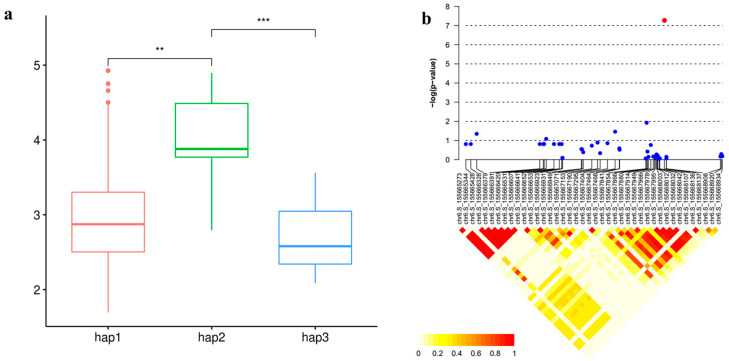
*GRMZM2G440968* affect Hg content in maize axis. (**a**). Haplotype analysis of *GRMZM2G440968*. Significance levels: ** *p* < 0.01, *** *p* < 0.001. (**b**). Expanded Manhattan plots showing the main SNPs, with the red dot indicating the lead SNP. R^2^ values of SNPs associated with *GRMZM2G440968* are also depicted.

Our exploration into candidate genes within the co-located loci unveiled several noteworthy findings (Table 1). For example, on chromosome 6, *GRMZM2G440968* encodes a cysteine proteinase inhibitor, which may alleviate mercury toxicity by inhibiting cysteine, as indicated by its known role in detecting kidney damage [37,38]. Moving to chromosome 10, *GRMZM2G005633* encodes Endochitinase B, a pivotal player in bacterial adaptation to stresses, promoting plant growth and development [39]. *GRMZM2G125991* encodes endoglucanase 7-like, a cellulose-related enzyme potentially involved in responding to copper pollution in willow roots, impacting cellulase content [40]. *GRMZM2G125943* encodes a histidine kinase, recognized for its pivotal role in plant stress and hormone regulation. Previous studies have illuminated its involvement in responding to challenges such as cold stress and Cordyceps sinensis mimicry [41,42]. An intriguing observation comes from a locus (the peak SNP is chr5.S_2356674) on chromosome 5, exclusively detected in the XX environment, housing seven candidate genes. Notably, *GRMZM2G002805*, *GRMZM2G144188* and *GRMZM2G144172* within this locus encode zinc finger proteins, known to play a role in alternative splicing under conditions of arsenic poisoning. Specifically, As^3+^ can displace Zn^2+^ in *ZRANB2*, leading to structural changes affecting protein function. Studies have also highlighted the impact of Hg (II) leading to structural changes affecting protein function [43,44,45,46]. Subsequently, 19 candidate genes were identified in 9 QTL, involved in protein synthesis and lipid metabolism. Further functional analysis, combining expression GWAS with the expression levels of 16 genes and 1.25 M SNPs, revealed significant correlations for all 16 genes (Table 1). Haplotype analysis of *GRMZM2G440968* demonstrated three haplotypes, with Hap3 exhibiting the lowest mercury content compared to other haplotypes. LD analysis within one LD decay distance (±30 kb) upstream and downstream of the lead SNP unveiled linkage relationships between them, emphasizing co-inheritance and linkage on the genome (Figure 6). These findings provide valuable insights into the genetic mechanisms underlying various physiological and metabolic processes in maize.

## 4. Discussion

In plant GWAS studies, maximizing the statistical power is paramount. Different maize traits have varying sensitivities to different statistical models, a phenomenon intricately linked to population structure. Yang’s seminal work underscored the pivotal role of selecting the most appropriate statistical model to augment GWAS statistical power [24]. Extending this line of inquiry, Zhao et al. conducted a GWAS study on arsenic content across five maize tissues, meticulously assessing different statistical models to balance minimizing false positives and optimizing analysis sensitivity. Their findings illuminated that the K model and Q + K model exhibited excessive conservatism, potentially increasing type II errors. In contrast, the Q model emerged as the optimal choice, effectively controlling false positives while preserving power [18].

In the present study, we encountered a similar scenario where both the K model and Q + K model demonstrated unwarranted stringency, while the Q model provided an ideal balance. Therefore, we selected the Q model to investigate the genetic basis of Hg content in five maize tissues. Using this model with 0.55 M SNPs, we identified more significant SNPs and loci compared to the previous study. However, increasing marker density and population size can further enhance GWAS power, as demonstrated by others [24,47]. To explore this, we reanalyzed the data using 1.25 M SNPs under the Q model. Directly comparing the results revealed substantial gains in power, with over double the number of significant SNPs and loci detected. This increase derives from a richer representation of the genome’s genetic variation, consistent with prior reports. Innovative statistical models have also proven effective by optimizing genetic and pedigree relationships [48]. Thus, both expanding genotype data and refining statistical models contribute synergistically to the increased statistical power of GWAS.

Analysis of candidate genes revealed several with notable functions. For example, *GRMZM2G162413* plays a key role in resisting ear rot and temperature stress [49]. Another significant candidate, *GRMZM2G011520* encodes arsenate reductase critical for arsenic detoxification [50]. *GRMZM5G877941* links to growth inhibition and senescence under methylglyoxal stress [51]. *GRMZM2G150496* exhibits hypersensitivity to arsenate stress and reduced uptake compared to wild-type [52]. The emerging candidate *GRMZM2G440968* encodes a cysteine proteinase inhibitor that may alleviate mercury toxicity by inhibiting cysteine, as indicated by its role in detecting kidney damage and ameliorating nephrotoxicity. We speculate that plants may undergo oxidative stress under mercury stress, leading to the production of reactive oxygen species and causing damage to cellular components. Cysteine proteinase inhibitors, by regulating the activity of cysteine proteinases, may help modulate the plant’s response to oxidative stress and maintain cellular homeostasis under mercury exposure (Supplementary Figure S6).

Mercury poisoning poses a substantial threat, with potential exposure pathways including ingestion through the food chain or contact with mercury-containing air, water, or specific occupational settings, leading to adverse effects on the nervous system [8]. Intriguingly, research indicates that even mercury levels below 50 µg/g in hair can pose neurological risks, manifesting as sensory disturbances [53]. Furthermore, studies underscore the neurobehavioral and neurochemical toxicity associated with prolonged low-dose cinnabar (HgS) exposure, shedding light on the sedative and neurotoxic effects of this mineral medicine [54]. The primary approach to mitigating mercury poisoning involves chelation therapy, designed to enhance methylmercury excretion by forming complexes with mercury ions. However, existing chelating agents have limitations, including reduced therapeutic efficacy and the potential loss of essential elements like iron and calcium. Some chelating agents may even exhibit toxicity [55,56]. Consequently, effective mercury poisoning treatment remains a formidable challenge. Amid these challenges, the cultivation of mercury-tolerant maize emerges as a promising solution to address mercury poisoning. Additionally, exploration of organic selenium compounds and thiourea resin has shown promise in reducing methylmercury toxicity. However, these agents primarily focus on excretion rather than tissue repair. Research has also spotlighted the beneficial effects of natural plant extracts in mitigating the toxic impact of methylmercury and reducing metal neurotoxicity. Notably, garlic’s mercaptans bind with methylmercury, reducing its toxicity. Fisetin, derived from various fruits, effectively reduces brain methylmercury accumulation and related toxicity. These findings open avenues for exploring natural substances in maize with therapeutic potential against mercury poisoning [57,58].

## 5. Conclusions

In this study, we leveraged an enlarged SNP panel comprising 1.25 million SNPs and improved statistical models to re-conduct a genome-wide association study of mercury content in five tissues of 230 maize inbred lines. Compared to prior research utilizing lower-density markers, our approach identified considerably more significant quantitative trait loci associated with mercury accumulation. Specifically, we uncovered 74 additional QTL, for a total of 169 candidate genes implicated in mercury content, of which 142 have annotated functions. Notably, *GRMZM2G440968*, encoding a cysteine proteinase inhibitor, emerges as a potential key regulator in alleviating mercury toxicity in plants. By inhibiting cysteine proteinases, this gene may help mitigate the strong binding of mercury to cysteine thiolate anions in vital enzymes. Haplotype analysis showed that lines containing haplotype 3 exhibited significantly lower mercury levels. Through enhanced marker density and improved modeling, our research provides deeper insights into the genetic mechanisms underlying mercury accumulation in maize. Ultimately, these findings are critical for developing maize varieties with reduced mercury content, thereby contributing to broader efforts at ensuring food safety and human health. In conclusion, we demonstrate that augmenting GWAS approaches can uncover additional genetic factors influencing metal stress tolerance in plants.

## Figures and Tables

**Table 1:** Candidate genes revealed by multiple locations.

ID ^a^	Chr.	Position ^b^	Trait	Location	SNP	*p* Value ^c^	R^2 d^	Candidate Gene ^e^	Annotation	Expressed or Not ^f^	Verified by Expression GWAS ^g^
1	4	167403585	Axis	BLUP	chr4.S_167403585	1.54 × 10^−6^	0.18	*GRMZM2G125943*	histidine kinase	express	Ture
								*GRMZM2G125991*	endoglucanase 7-like	express	Ture
								*GRMZM2G029859*	pentatricopeptide repeat-containing protein At3g16610	express	Ture
2	4	167403585	Axis	XX	chr4.S_167403585	1.33 × 10^−6^	0.18	*GRMZM2G125943*	histidine kinase	express	Ture
								*GRMZM2G125991*	endoglucanase 7-like	express	Ture
								*GRMZM2G029859*	pentatricopeptide repeat-containing protein At3g16610	express	Ture
3	5	2356674	Kernel	XX	chr5.S_2356674	1.31 × 10^−6^	0.10	*GRMZM2G002825*	actin-depolymerizing factor 3	express	Ture
								*GRMZM2G002805*	zinc finger protein ZAT5	express	Ture
								*GRMZM2G002815*	NA	express	Ture
								*GRMZM2G144188*	dof zinc finger protein DOF2.4-like	not	
								*GRMZM2G144172*	dof zinc finger protein DOF2.4-like	express	Ture
								*GRMZM2G003068*	NA	express	Ture
								*GRMZM2G003108*	CRAL/TRIO domain containing protein	express	Ture
4	6	155668107	Axis	BLUP	chr6.S_155668107	2.82 × 10^−8^	0.13	*GRMZM2G566873*	NA	not	
								*GRMZM2G140805*	NA	not	
								*GRMZM2G440949*	dr1-associated corepressor	express	Ture
								*GRMZM2G440968*	cystatin 3	express	Ture
								*GRMZM2G140817*	putative cytochrome P450 superfamily protein	express	Ture
5	6	155668107	Axis	CG	chr6.S_155668107	6.77 × 10^−7^	0.10	*GRMZM2G566873*	NA	not	
								*GRMZM2G140805*	NA	not	
								*GRMZM2G440949*	dr1-associated corepressor	express	Ture
								*GRMZM2G440968*	cystatin 3	express	Ture
								*GRMZM2G140817*	putative cytochrome P450 superfamily protein	express	Ture
6	6	155668107	Axis	XX	chr6.S_155668107	1.46 × 10^−8^	0.13	*GRMZM2G566873*	NA	not	
								*GRMZM2G140805*	NA	not	
								*GRMZM2G440949*	dr1-associated corepressor	express	Ture
								*GRMZM2G440968*	cystatin 3	express	Ture
								*GRMZM2G140817*	putative cytochrome P450 superfamily protein	express	Ture
7	10	127359876	Stem	BLUP	chr10.S_127359876	8.01 × 10^−9^	0.23	*GRMZM2G005633*	Endochitinase B	express	Ture
								*GRMZM2G006428*	NA	express	Ture
								*GRMZM2G006216*	S-adenosyl-L-methionine-dependent methyltransferase superfamily protein	express	Ture
								*GRMZM2G005939*	NA	express	Ture
8	10	127359876	Stem	CG	chr10.S_127359876	1.11 × 10^−6^	0.17	*GRMZM2G005633*	Endochitinase B	express	Ture
								*GRMZM2G006428*	NA	express	Ture
								*GRMZM2G006216*	S-adenosyl-L-methionine-dependent methyltransferase superfamily protein	express	Ture
								*GRMZM2G005939*	NA	express	Ture
9	10	127359876	Stem	XX	chr10.S_127359876	5.81 × 10^−9^	0.24	*GRMZM2G005633*	Endochitinase B	express	Ture
								*GRMZM2G006428*	NA	express	Ture
								*GRMZM2G006216*	S-adenosyl-L-methionine-dependent methyltransferase superfamily protein	express	Ture
								*GRMZM2G005939*	NA	express	Ture

Notes: All QTL with overlapping QTL regions were categorized as a locus. ^a^. ID of loci was numbered according to chromosome and position by an ascending order method; ^b^. Physical position of each SNP based on B73 RefGen_v2; ^c^. *p* value of the corresponding trait calculated by Q model; ^d^. The phenotypic variance explained by the corresponding locus; ^e^. A plausible biological candidate gene in the locus or the nearest annotated gene to the lead SNP. ^f^. Whether the gene is expressed or not; ^g^. validation of the correlation analysis of gene expression level.

## Data Availability

The datasets supporting the conclusions of this article are included within the article (and its supplementary files). Genotypic data could download from http://www.maizego.org/Resources.html (accessed on 18 February 2024), and the phenotypic data could obtain from reference [27] or be shared on reasonable request of the corresponding authors.

## Supplementary Materials

The following supporting information can be downloaded at: https://www.mdpi.com/article/10.3390/genes15020257/s1, Table S1. Pedigree information of the 230 inbred lines used in this study. Table S2. List of candidate genes and their annotations. Figure S1. Comparison of Manhattan plots resulting from GWAS, based on 0.55 M SNPs, using Q model and 5PCs + K model for mercury content in maize axis, stem, bract, leaf, and kernel at BLUP environments. Figure S2. Comparison of quantile–quantile (QQ) plots resulting from GWAS, based on 0.55 M SNPs, using three models (Q, K, and Q + K) for mercury content in maize axis, stem, bract, leaf, and kernel in three environments. Figure S3. Comparison of quantile–quantile (QQ) plots resulting from GWAS, based on 1.25 M SNPs, using three models (Q, K, and Q + K) for mercury content in maize axis, stem, bract, leaf, and kernel in three environments. Figure S4. Comparison of quantile–quantile (QQ) plots resulting from GWAS, based on 0.55 M SNPs and 1.25 M SNPs, using Q model for mercury content in maize axis, stem, bract, leaf, and kernel in three environments. Figure S5. Comparison of Manhattan plots resulting from GWAS, based on 0.55 M SNPs and 1.25 M SNPs, using Q model for mercury content in maize axis, stem, bract, leaf, and kernel in three environments. Figure S6. Schematic representation of *GRMZM2G440968* modulating cysteine protease in response to oxidative stress.
