# Supplementary material for: Identification of Novel QTL for Mercury Accumulation in Maize Using an Enlarged SNP Panel
Source: Genes (Basel). 2024 Feb 19;15(2):257. doi: 10.3390/genes15020257 (PMC10888321; doi:10.3390/genes15020257)
Supplement: Supplementary file 1 [file genes-15-00257-s001.zip › Supplementary Figures.pdf]

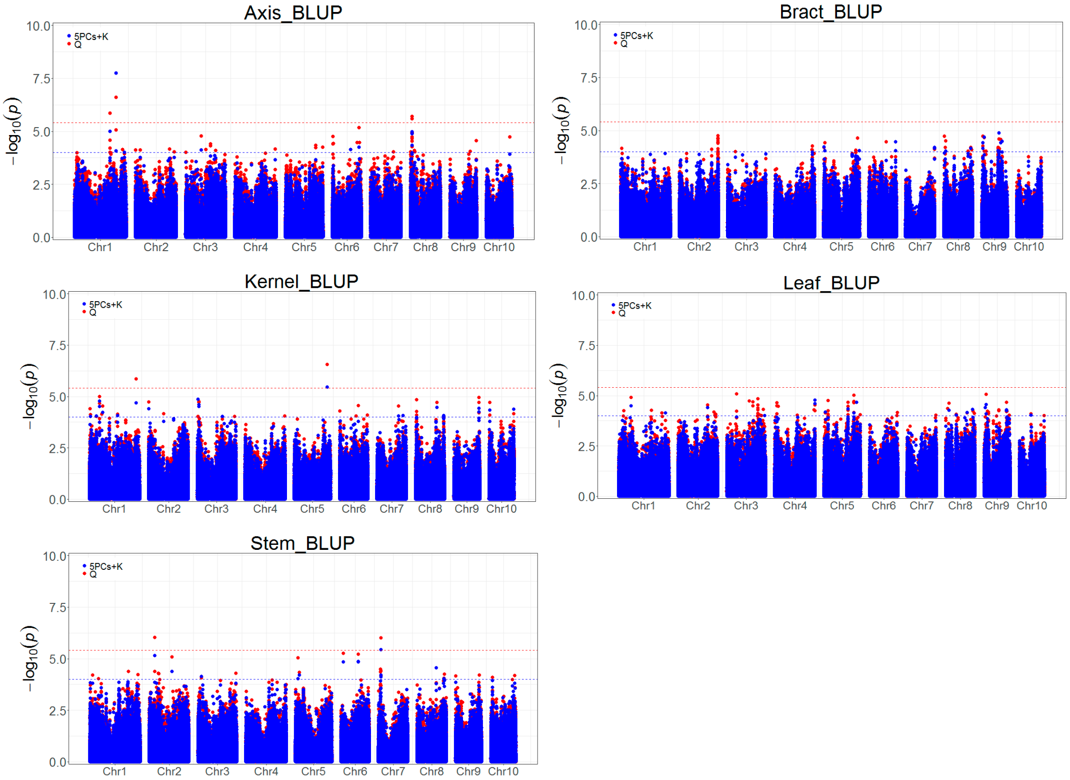

Figure S1. Comparison of Manhattan plots resulting from GWAS, based on 0.55 M SNPs, using Q model and 5PCs + K model for mercury content in maize axis, stem, bract, leaf, and kernel at BLUP environments.

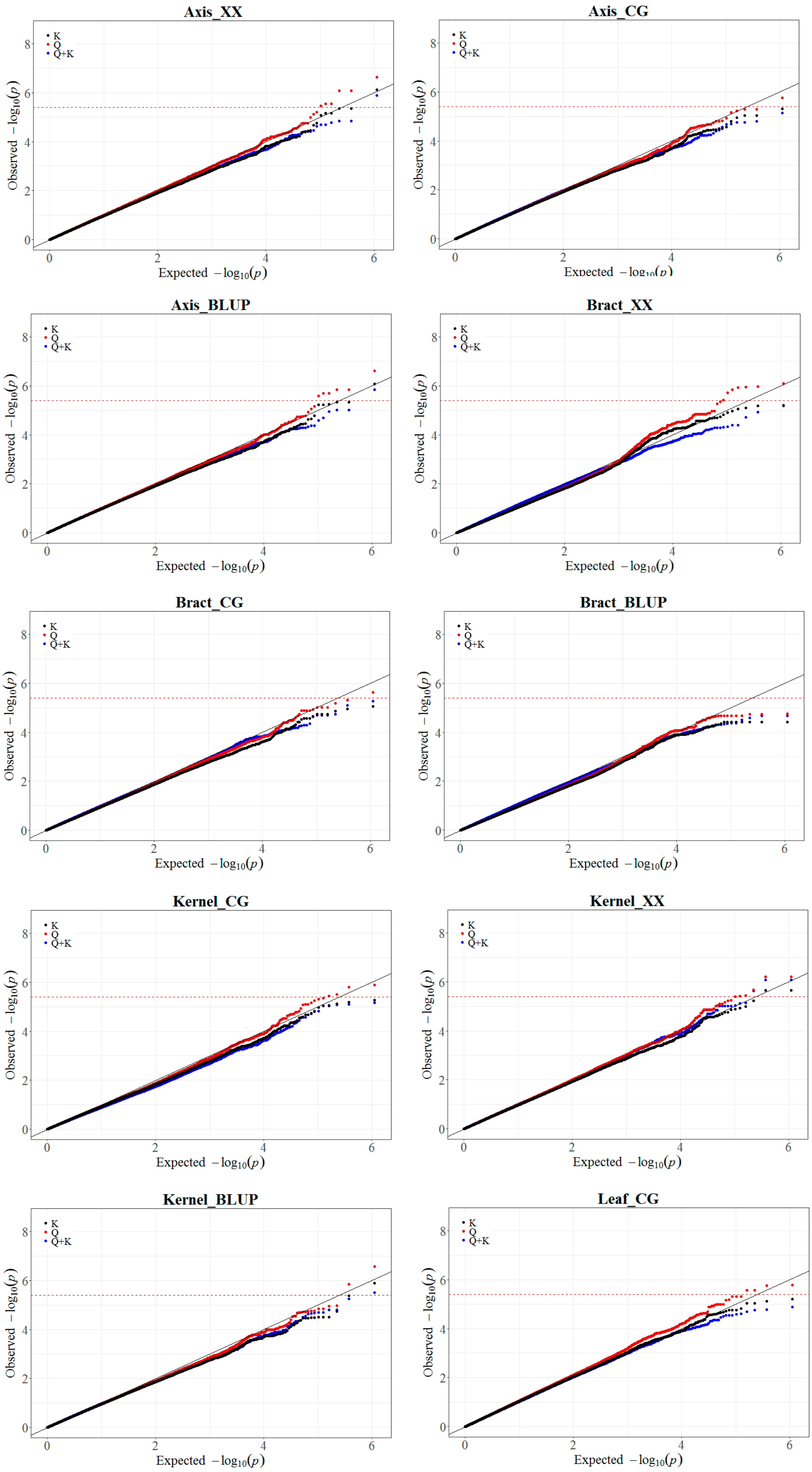
Axis_XX
Axis_CG
Axis_BLUP
Bract_XX
Bract_CG
Bract_BLUP
Kernel_CG
Kernel_XX
Kernel_BLUP
Leaf_CG
K
Q
Q+K
Observed $-\log_{10}(p)$
Expected $-\log_{10}(p)$
0
2
4
6
8

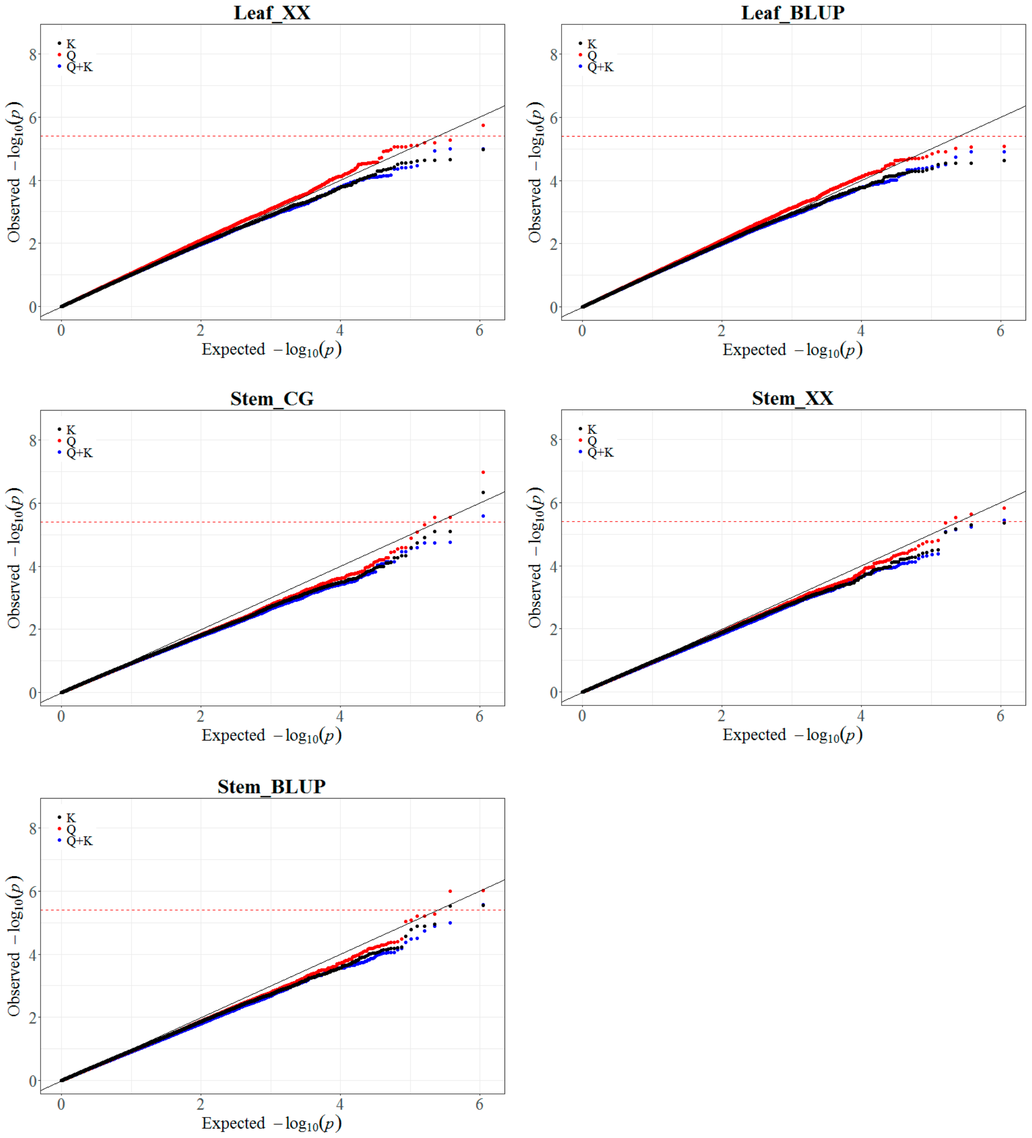

Figure S2. Comparison of quantile–quantile (QQ) plots resulting from GWAS, based on 0.55 M SNPs, using three models (Q, K, and Q + K) for mercury content in maize axis, stem, bract, leaf, and kernel in three environments.

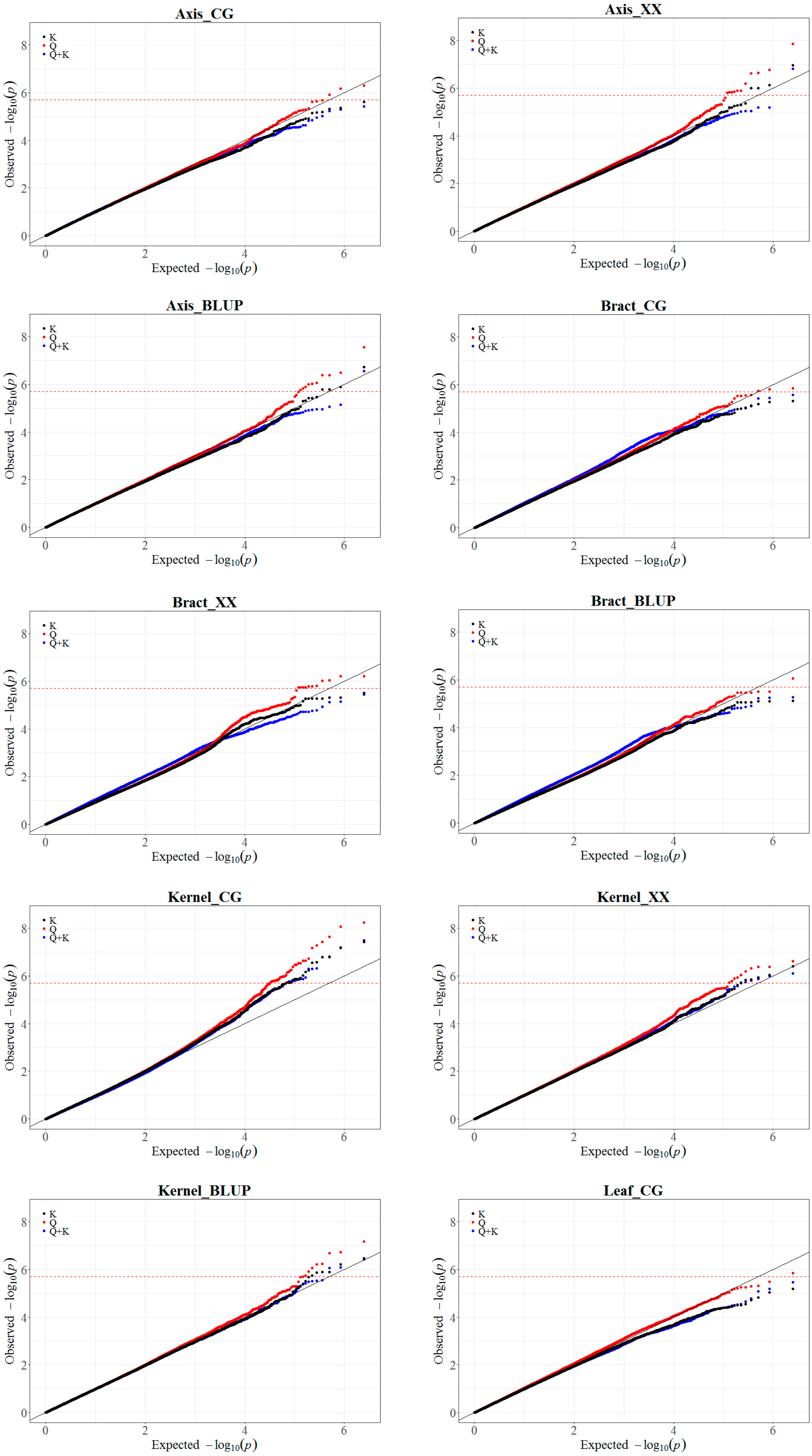
Axis_CG
K
Q
Q+K
Observed −log10(p)
Expected −log10(p)
Axis_XX
K
Q
Q+K
Observed −log10(p)
Expected −log10(p)
Axis_BLUP
K
Q
Q+K
Observed −log10(p)
Expected −log10(p)
Bract_CG
K
Q
Q+K
Observed −log10(p)
Expected −log10(p)
Bract_XX
K
Q
Q+K
Observed −log10(p)
Expected −log10(p)
Bract_BLUP
K
Q
Q+K
Observed −log10(p)
Expected −log10(p)
Kernel_CG
K
Q
Q+K
Observed −log10(p)
Expected −log10(p)
Kernel_XX
K
Q
Q+K
Observed −log10(p)
Expected −log10(p)
Kernel_BLUP
K
Q
Q+K
Observed −log10(p)
Expected −log10(p)
Leaf_CG
K
Q
Q+K
Observed −log10(p)
Expected −log10(p)

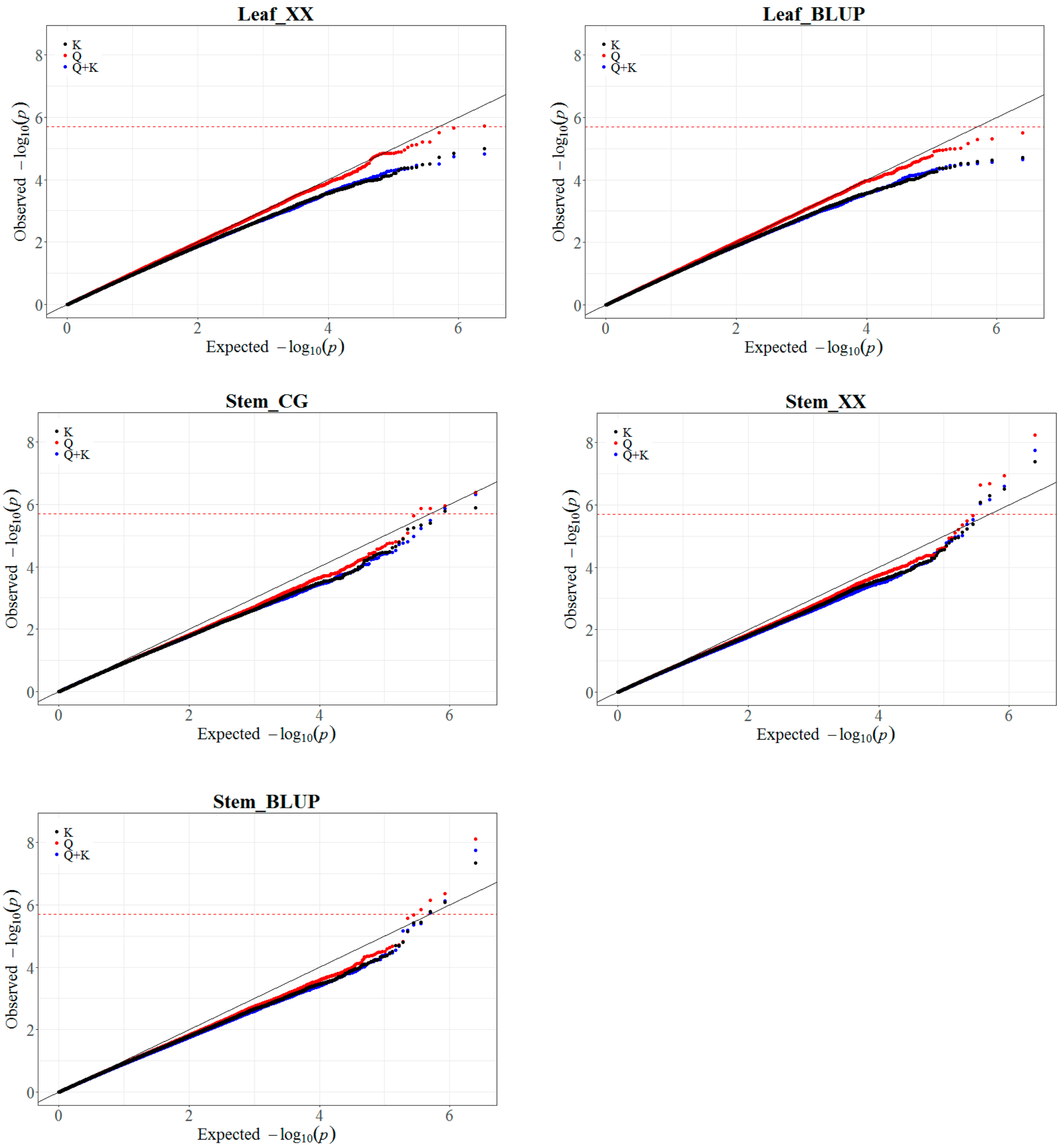

Figure S3. Comparison of quantile–quantile (QQ) plots resulting from GWAS, based on 1.25 M SNPs, using three models (Q, K, and Q + K) for mercury content in maize axis, stem, bract, leaf, and kernel in three environments.

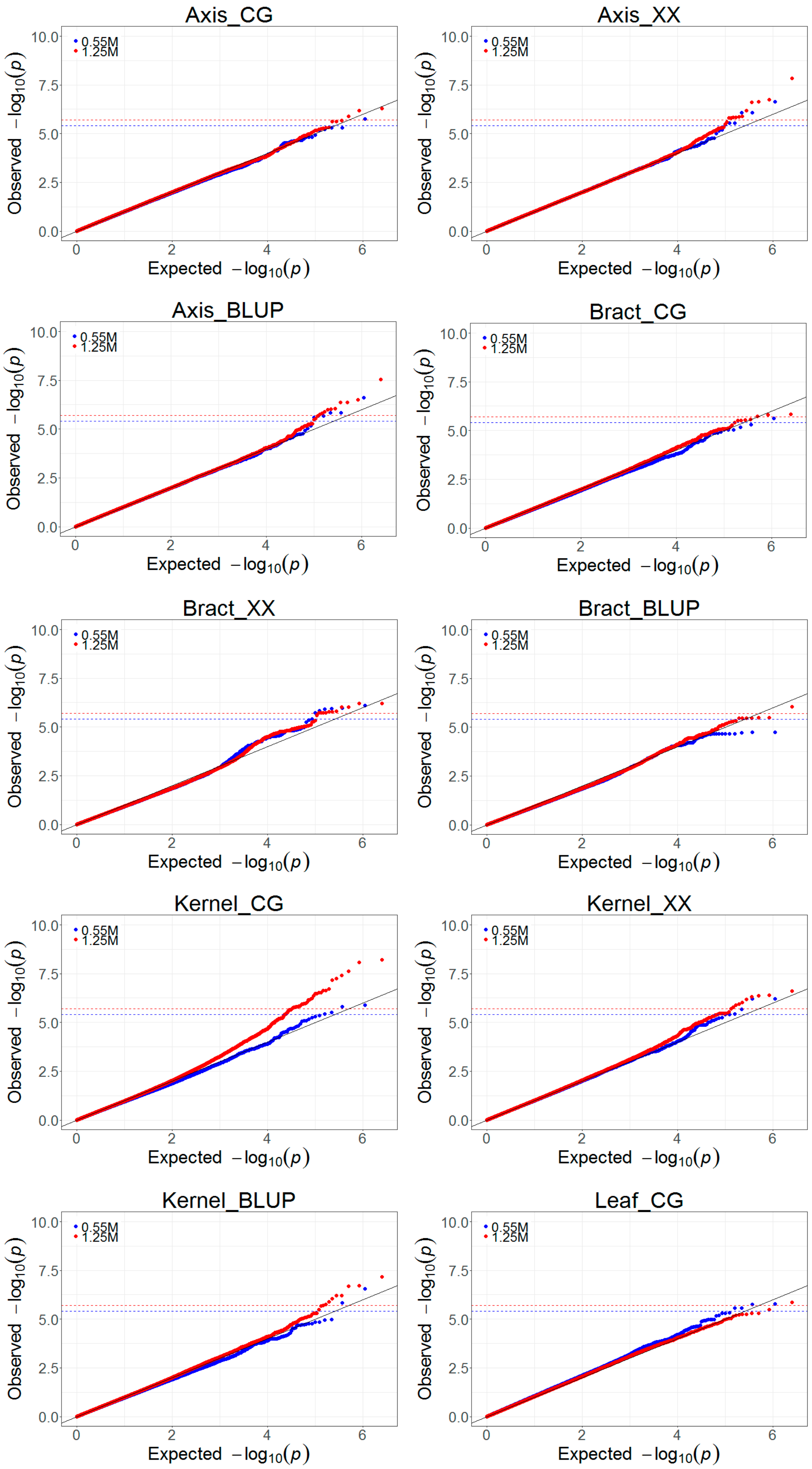

Axis_CG
Axis_XX
Axis_BLUP
Bract_CG
Bract_XX
Bract_BLUP
Kernel_CG
Kernel_XX
Kernel_BLUP
Leaf_CG
0.55M
1.25M
Observed $-\log_{10}(p)$
Expected $-\log_{10}(p)$
0.0
2.5
5.0
7.5
10.0
0
2
4
6

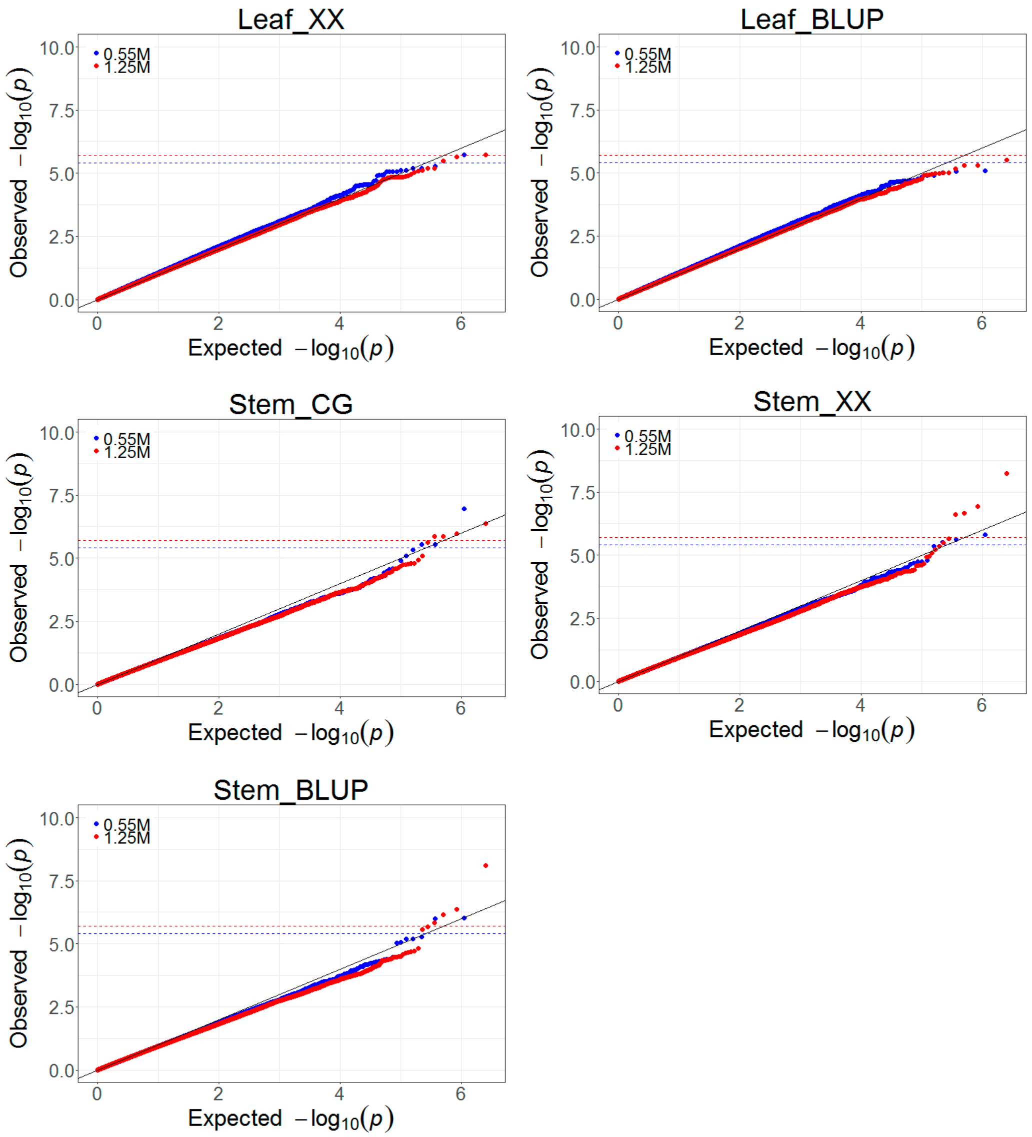

Figure S4. Comparison of quantile–quantile (QQ) plots resulting from GWAS, based on 0.55 M SNPs and 1.25 M SNPs, using Q model for mercury content in maize axis, stem, bract, leaf, and kernel in three environments.

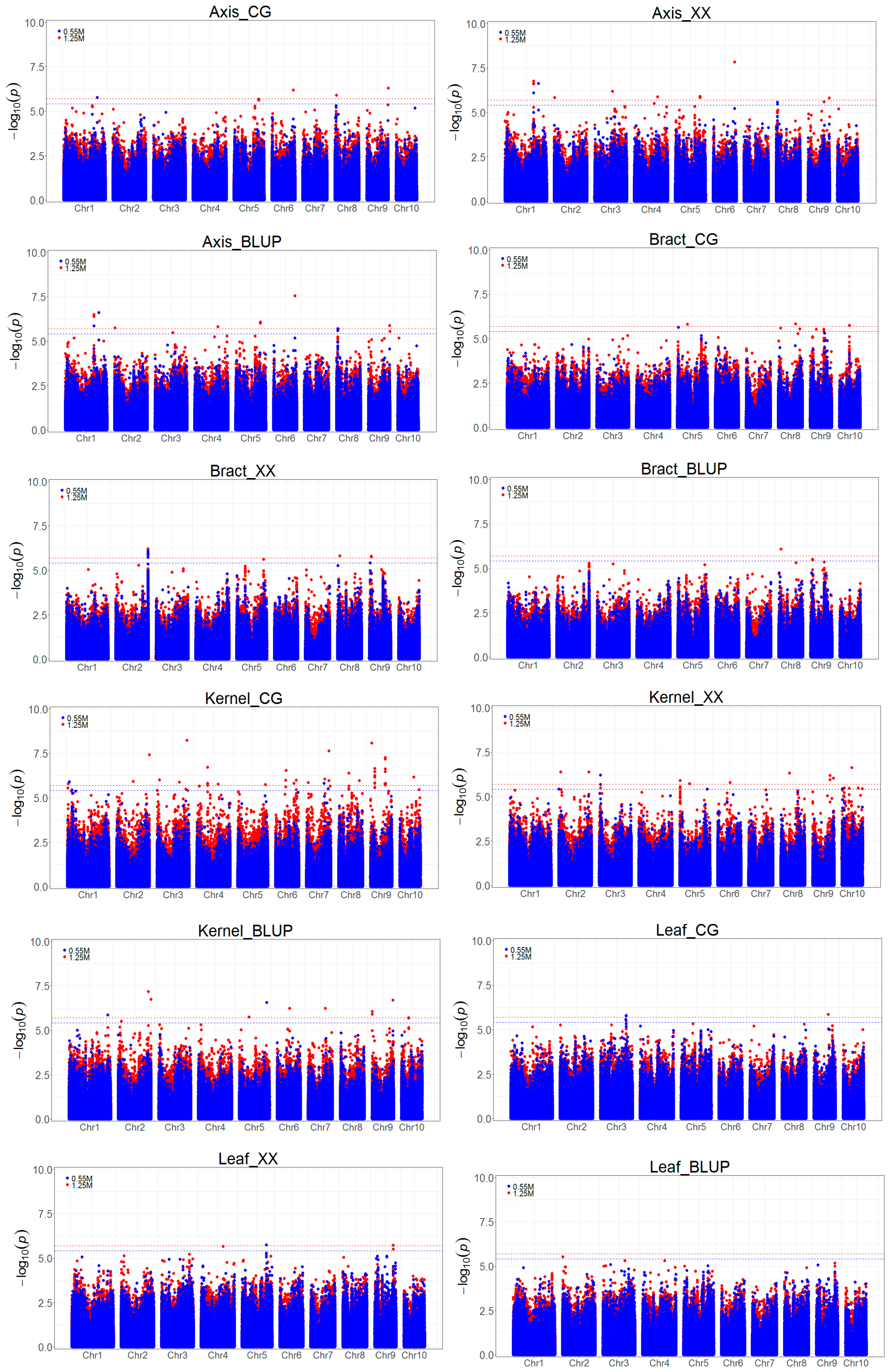

Axis_CG
Axis_XX
Axis_BLUP
Bract_CG
Bract_XX
Bract_BLUP
Kernel_CG
Kernel_XX
Kernel_BLUP
Leaf_CG
Leaf_XX
Leaf_BLUP
0.55M
1.25M
$-\log_{10}(p)$
0.0
2.5
5.0
7.5
10.0
Chr1 Chr2 Chr3 Chr4 Chr5 Chr6 Chr7 Chr8 Chr9 Chr10

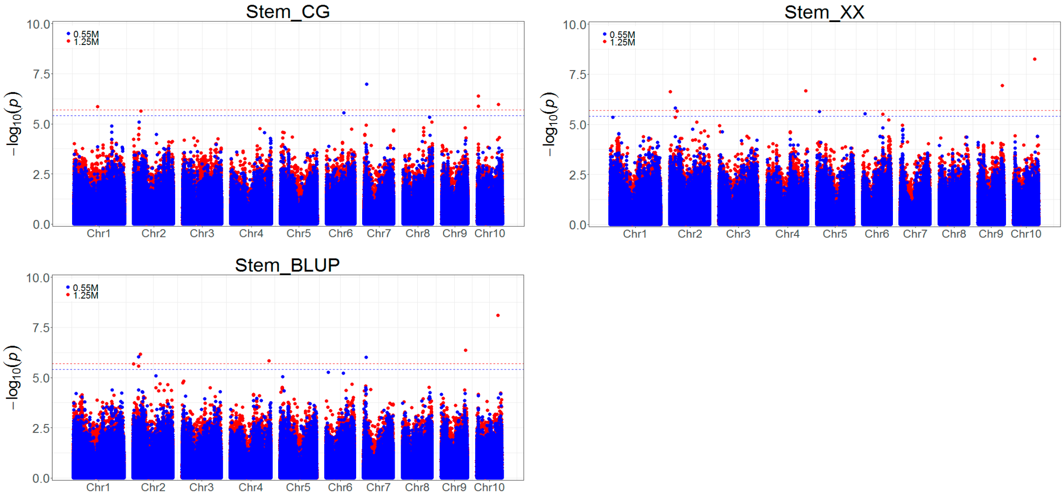

Figure S5. Comparison of Manhattan plots resulting from GWAS, based on 0.55 M SNPs and 1.25 M SNPs, using Q model for mercury content in maize axis, stem, bract, leaf, and kernel in three environments.

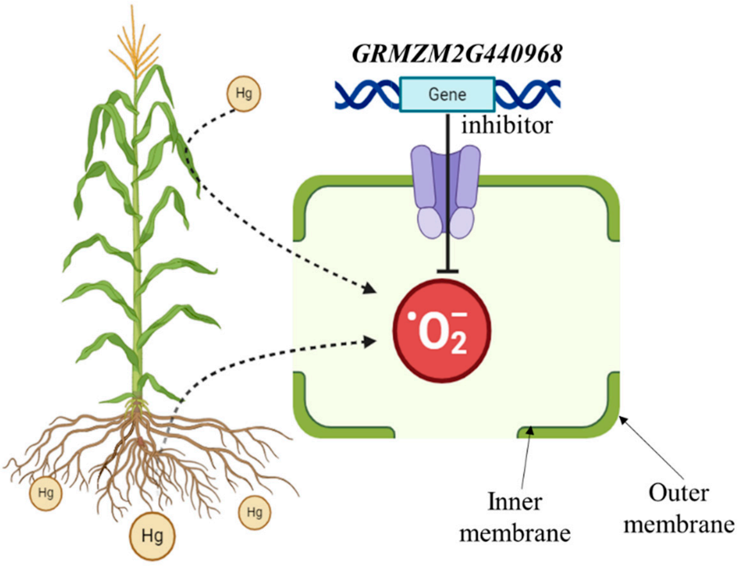

Figure S6. Schematic representation of GRMZM2G440968 modulating cysteine protease in response to oxidative stress.
